# “I’m Too Old for That”: The Role of Ageism and Sexual Dysfunctional Beliefs in Sexual Health in a Sample of Heterosexual and LGB Older Adults: A Pilot Study

**DOI:** 10.3390/healthcare11040459

**Published:** 2023-02-05

**Authors:** Luca Flesia, Merylin Monaro, Emmanuele A. Jannini, Erika Limoncin

**Affiliations:** 1 Foster Care and Family Solidarity Unit, Azienda ULSS6 Euganea, Via Degli Scrovegni, 14, 35131 Padova, Italy; 2 Department of General Psychology, University of Padova, 35122 Padova, Italy; 3 Endocrinology and Medical Sexology (ENDOSEX), Department of Systems, Medicine, University of Rome Tor Vergata, 00133 Rome, Italy; 4 Department of Dynamic, Clinical Psychology and Health Sciences, Sapienza University of Rome, 00185 Rome, Italy

**Keywords:** ageism, sexual health, sexuality, LGB, gay, beliefs, ageing, elderly, older adults, stereotypes

## Abstract

The role of ageism (i.e., discrimination against individuals or groups on the basis of their age), in particular toward the sexuality of the elderly, remains, to date, an overlooked issue. A few studies have suggested that ageistic stereotypes can negatively affect older adults’ sexual health. No data are available, in particular, about differences among heterosexual and LGB (lesbian, gay, and bisexual) populations. The present study aimed to investigate differences in perceived ageism and related dysfunctional beliefs in a sample of heterosexual (*n* = 104) and LGB (*n* = 103) older adults (aged 55 or older; mean age 66.5) and their impact on sexual health and satisfaction. LGB individuals reported higher rates of masturbation and sexual intercourse and an increased quality of sexual activity as compared to heterosexuals. In addition, no differences between the groups emerged as regards perceived ageism and dysfunctional beliefs toward ageing. In conclusion, LGB individuals perceived more ageism toward sexuality than their counterparts; however, heterosexuals were more likely to have dysfunctional beliefs toward sexuality in ageing. The study findings highlight the significance of examining sexual orientation to understand experiences of sexuality in ageing of the growing older population. Renewed socio-educational efforts based on these data are clearly needed.

## 1. Introduction

The age of the world population is increasing. It is estimated that by 2050 nearly one in five people will be over the age of 60 [1].

Despite this, today’s society is still significantly permeated by an “ageing-phobic” vision that unfairly tends to depict and lump all people aged 65 or older into a group of old, frail, undesirable, and asexual beings, separate from the rest of society [2]. Such stereotypical representations are embodied and internalized since childhood and can negatively impact people’s health while ageing under many aspects, including sexual health [3].

Ageism refers to the stereotypes (how we think), prejudice (how we feel), and discrimination (how we act) directed towards individuals or groups based on their age. It can be institutional, interpersonal, or self-directed (“internalized ageism”) [4,5]. Ageism toward older people is known to seriously affect their lives in terms of both health and well-being. For older people, ageism is associated with poorer physical health [6], worse mental health [7], and a shorter lifespan [8]. It leads to a poorer quality of life [9] and higher rates of loneliness and social isolation [10], therefore representing a significant obstacle to so-called “successful ageing” [11].

Sexual well-being, according to the World Health Organization (WHO), refers specifically to the absence of disease and coercion and draws attention to sexual rights and to the possibility of sexual pleasure [12]. Recently, studies have begun to recognize sexual well-being as a lifelong consideration as well as a multidimensional construct [12]. With regard to sexual well-being and activity in later life, ageism certainly represents a significant threat to sexual health and sexual satisfaction among older adults. Although a growing abundance of evidence shows that older adults are sexually active [13,14], older people are often stereotyped as non-sexual beings who should not, cannot, and do not want to have sexual relationships. Therefore, sexuality in later life is dangerously underrepresented [14] and older people’s sexual needs are rarely addressed [13], contributing to poor sexual and reproductive health and increased rates of sexually transmitted diseases (STDs) among older adults [15,16].

To date, few studies have investigated the role of ageism in sexual health. Research shows that older people often internalize ageist stereotypes and myths regarding sexuality in later life, taking on dysfunctional beliefs about sexuality. Dysfunctional beliefs about sexuality are defined as rigid, negative thoughts about sexual habits and sexual life that influence personal self-perception of sexual desire and sexual arousal [17]. Specifically, as people age, stereotypes regarding sexuality and ageing, internalized since childhood and reinforced during adulthood, become self-stereotypes, therefore creating a vicious circle between dysfunctional beliefs and sexual activity. Of note, the literature reports that they often work below self-awareness [18,19].

Older people are reluctant to express their sexuality and are often hesitant to discuss sexual issues with doctors for fear of being met with disapproval [20]. Heywood et al. explored the possible links between experiences of ageism and sexual activity/interest in later life among Australians over 60; the authors found that experiences of ageism were more likely to be reported by those who had not had sex in the past two years and were not sure about their hopes/plans for sex in the future [21]. Those who reported that their sexual interest had increased or decreased since 60 also reported greater levels of ageism experience, as did those who wanted to have sex more frequently in the future. Sym and Cohn, analyzing the impact of aging sexual stigma on sexual and intimate activity among midlife and older adults (aged 50 or older), found that ageist sexual stereotypes affect individual sexual health and wellness via internalized beliefs (not via gender or age cohort), predicting lower engagement in both sexual and intimate activities [22]. Graf and Patrick obtained similar results within a sample of midlife adults (aged 45 or older) [23].

Of note, none of the studies considered sexual orientation as a study variable. Although this has been a neglected issue, differentiating research on ageism and sexuality in ageing according to sexual orientation could be a significant aspect to investigate. Indeed, the literature indicates that the LGB (lesbian, gay, and bisexual) community, as a stigmatized population, undergoes specific structural, interpersonal, and individual stressors, that can specifically and uniquely impact LGB people’s mental health and physical health, as well as their social health [24]. Moreover, ageism is reported to interact with other forms of bias, such as sexism and ableism, therefore exacerbating their negative impact on personal health and well-being, as well as compounding their disadvantage on specific vulnerable populations [25,26]. Therefore, the intersection between ageism and heterosexism and sexuality is one that could be worth exploring.

Of note, research on ageism and the LGB population is not only still very scant but the literature regarding LGB people’s ageing and LGB people’s sexuality in ageing is also poor. Such paucity of research in the field likely mirrors the so-called “double invisibility” the population of lesbian, gay, and bisexual older people suffer because of a double stigmatization process: being old and belonging to a non-normative sexual minority. Research investigating the issue of LGB older adults‘ sexual health suggests that LGB older adults tend to experience significantly lower sexual satisfaction and more sexual problems than their counterparts [13,27]; internalized homo/biphobia and the need to conceal personal sexual identity (sexuality acceptance concerns) are reported to be connected to low levels of sexual satisfaction [28,29]. Moreover, assumed as heterosexual, LGB older adults must face the dilemma of hiding their sexual identity or risking discrimination in health provisions [14].

Sexual life is relevant at every stage of life and impacts on people’s well-being and general health; ageism and dysfunctional beliefs toward ageing and sexuality in ageing impact on people’s well-being and general health as well; to date, no investigations have compared these issues between the heterosexual and the LGB population. Given all these considerations, the aim of the present study is to investigate the role of ageism toward ageing and sexuality in ageing on heterosexual and LGB older adults’ sexual health. Specifically, we aimed to investigate differences between the study groups in terms of the presence of perceived social ageism, ageism toward sexuality in the elderly, and dysfunctional beliefs related to the elderly and to the sexuality of the elderly.

## 2. Materials and Methods

### 2.1. Participants and Procedure

Data were collected cross-sectionally through an online survey managed using Google Forms between August 2021 and May 2022. The inclusion criteria were: being 55 years or older, living in Italy, and being a native Italian speaker. The definition of an “elderly person” was only partly based on the international criteria proposed by the World Health Organization (WHO), [30], as the previous literature reports that the physiological aspects related to sexuality start to decline from the age of 60 or lower [31]. The exclusion criteria were: being younger than 55; not being a native Italian speaker; and residing outside of Italy, because ageism may be culturally bound [32].

To recruit the LGB community sample, information about the study was disseminated through mailing lists and social media (i.e., Facebook and WhatsApp) thanks to a collaboration with Italian LGB associations and community centers (i.e., ArciGay associations), using convenience and snowball sampling techniques.

Heterosexual participants were recruited through the dissemination of the survey link on online platforms and social media (i.e., Facebook and WhatsApp) using convenience and snowball sampling techniques.

All of the participants were asked to fill out a questionnaire created ad hoc for this study that was aimed at specifically investigating ageism toward the elderly, ageism associated with sexuality, and dysfunctional beliefs associated with the elderly and with sexuality. The participants provided informed consent before filling out the questionnaire and were provided with guarantees about anonymity and confidentiality issues. Participation was voluntary; they did not receive any compensation for participation.

The current project was designed in accordance with the Declaration of Helsinki and was approved by the Ethical Committee for Psychological Research of the University of Padova (Unique Code 33DA01B8DA2FF80BD1D7A02EEB6B5AF1).

### 2.2. Measures

The online ad hoc questionnaire, administered in Italian, consisted of 22 multiple-choice questions. For the study purpose, the following information was investigated:

Socio-demographics: Sex assigned at birth (male or female), sexual orientation (heterosexual, bisexual, or homosexual), gender identity (cisgender or transgender/non-binary), age, relational status (celibate/nubile; civil marriage; marriage/cohabitation; separated; divorced; widowed), and religious belief (believer and practicing; believer and not practicing; non-believer)).

Physical and psycho-social well-being: Subjective perception about personal health status (bad; good; optimal), mood (to experience depression always, often, sometimes, rarely, or never), and loneliness (always, often, sometimes, rarely, or never) during the last 12 months and weekly frequency of participation in social activities.

Perceived ageism toward ageing and toward the sexuality of elders: The perception of ageistic social representation of ageing was measured through the following questions: “In your opinion, what representation does society give about elder people?” (the response options were: “Positive”, “Neither positive nor negative”, and “Negative”) and “Do you feel represented by the image society gives of elderly people?” (the response options were: “Not at all” “A little”, “Enough”, and “A lot”). The questions have been modified from the perceived ageism questionnaire [33]. Perception about the ageistic social representation of sexuality in ageing was measured through the following question/statement: “Society represents elders as asexual” (with responses on a five-point Likert scale from “Totally disagree” to “Totally agree”);

Dysfunctional beliefs toward ageing and toward sexuality in ageing: Concern about ageing was measured through the following question: “Are you worried about the idea of getting old?” (the response options were: “Not at all”, “A little”, “Enough”, and “A lot”); dysfunctional beliefs about sexuality in ageing were measured through the following questions/statements: “Sexuality is important at all life stages” (the response options were on a five-point Likert scale from “Totally disagree” to “Totally agree”) and “Sexuality is not about older people” (the response options were on a five-point Likert scale from “Totally disagree” to “Totally agree”). The questions about dysfunctional beliefs were modified from the attitude to ageing questionnaire [34].

Sexual activity: The participants were assessed for changes in the frequency of sexual activity in the last 12 months (i.e., frequency of masturbation and sexual intercourse), for changes in perceived quality of sexual activity, and for the presence of sexual dysfunctions in the last 12 months based on the DSM 5th edition [35], with the following statements/questions: “In the last 12 months, the frequency of sexual intercourse has increased” (the response options were on a five-point Likert scale from “Totally disagree” to “Totally agree”); “In the last 12 months, the frequency of masturbation has increased” (the response options were on a five-point Likert scale from “Totally disagree” to “Totally agree”); “As compared to when I was aged 20–40, the quality of my sexual activity has ameliorated” (the response options were on a five-point Likert scale from “Totally disagree” to “Totally agree”); “In the last 12 months, I have been suffering from one or more of the following sexual dysfunctions: erectile dysfunction, hypoactive sexual desire disorder, premature ejaculation, retarded ejaculation, sexual pain, orgasm disease, a genito-pelvic pain /penetration disorder, or a sexual interest/arousal disorder” (the response options were: “Yes”; “No”).

Note that two attention check questions were added throughout the questionnaire, in order to reveal an inaccurate response style (e.g., “Please select the option ‘strongly agree’ to show you are paying attention to this question”).

### 2.3. Data Analysis

All statistical analyses were performed by MEDCALC version 13 [36].

As concerns the sample size, the previous literature pointed out that there are no data regarding the total number of LGB older adults in Italy, due to the significant invisibility that characterizes this population [37,38]. Therefore, it is difficult to determine the sample size based on its representativeness of social distribution. In this study, the sample size was defined based on the literature evidence affirming that for pilot studies, a sample size of 12 subjects per group could be a good numerosity [39] or a range of 10 to 40 participants per group depending on the parameter of interest [40] or at least 50 participants [41]. Moreover, a power analysis was computed a priori to ensure sufficient statistical power of data analysis. It has been calculated that a sample size of 90 is sufficiently large to achieve a statistical power (1−β) = 0.95 in a multiple logistic regression analysis involving 17 predictors, given a significance level α = 0.05 and a medium effect size [42].

The continuous variables were normally distributed (Shapiro–Wilk test) and were presented as the means and standard deviation. Dichotomous variables were condensed as absolute and relative frequencies. Univariate logistic regression analyses were performed to evaluate the differences between the two study groups. Multiple logistic regression analyses were then performed to evaluate the possible impact of selected variables on the study outcomes, which showed statistically significant differences with univariate analysis: perceived social ageism toward the sexuality of elderly people; dysfunctional beliefs about the elderly; and dysfunctional beliefs about the sexuality of elderly people. An alpha error of 5% was required to reject the null hypothesis. The analysis was performed using the stepwise variable selection method.

## 3. Results

### 3.1. Demographic Information

A total of 244 respondents accessed the survey; 29 subjects were excluded for the following reasons: being under 18 years of age (*n* = 4), not having completed the questionnaire fully (*n* = 16), or having withdrawn their consent (*n* = 9).

All of the demographics of 215 valid responses, along with the results of the comparison for demographic variables between the two study groups, are reported in Table 1. The two samples show some differences, specifically in terms of gender distributions and relational status. With regard to gender identity, only eight respondents declared a “transgender/non-binary” gender identity. Given the specificity of this population and the very small number of transgender/non-binary respondents, we decided to exclude this sub-sample from further statistical analysis.

Therefore, the final analytic study groups were composed of 103 LGB males and females and 104 heterosexual males and females.

Table 2 reports the results related to physical and psycho-social well-being. Specifically, in terms of physical and psycho-social well-being, our samples reported a good level of subjective perception of health and declared to experience depression and loneliness in the past 12 months in a moderate manner, without significant differences between heterosexual and LGB people.

### 3.2. Sexual Activity

Table 3 shows the results of the sexual activity of heterosexuals and LGB elderly people. With univariate logistic regression analysis, the following variables resulted in statistically significant differences between the two study groups; the LGB participants reported higher rates of increased frequency of sexual intercourse (*p* = 0.0009), increased frequency of masturbation (*p* = 0.001), and increased quality of sexual intercourse (*p* = 0.001). For the heterosexual group, multiple logistic regression showed that: (1) the experience of depression (OR = 0.33; 95% CI 0.155 to 0.993; *p* = 0.048) and the perception of the improvement of the quality of sexual intercourse (OR = 3.52; 95% CI 1.13 to 10,89; *p* = 0.02) were significantly associated with the frequency of sexual intercourse in the last 12 months; (2) perceived ageism significantly impacted on the frequency of masturbation (OR = 6.84; 95% CI 1.36 to 34,26; *p* = 0.01); (3) dysfunctional beliefs about the elderly significantly negatively impacted on the perception of the quality of sexual intercourse (OR = 0.08; 95% CI 0.01 to 0.63; *p* = 0.01). For the LGB group, multiple logistic regression showed that (1) cohabitation status (OR = 8.79; 95% CI 2.41 to 31.97; *p*= 0.001) and participation in social activities (OR = 17.67; 95% CI 3.61 to 86.48; *p* = 0.03) were significantly associated with the frequency of sexual intercourse in the last 12 months; perceived social ageism toward sexuality in elderly people (OR = 4.55; 95% CI 1.75 to 11.85; *p* = 0.01) and cohabitation status (OR = 0.33; 95% CI 0.12 to 0.94; *p* = 0.03) were significantly associated with the frequency of masturbation; the occasions of sociality were significantly associated with the perception of the quality of sexual intercourse (OR = 3.82; 95% CI 1.27 to 11.47; *p* = 0.01), (Supplementary Tables S1–S6).

### 3.3. Perceived Ageism toward Ageing and Sexuality

Table 4 reports the results regarding perceived social ageism toward ageing in the two study populations. The data highlighted that the two groups did not significantly differ (*p* > 0.05) as regards perceived social ageism toward ageing: specifically, 57.7% of the sample both of the heterosexual individuals and 54.5% of the sample of LGB individuals reported the presence of perceived ageism toward ageing.

Table 5 shows the results regarding perceived social ageism toward sexuality in ageing in the two groups. The LGB participants reported significantly higher levels of such types of ageism than their counterparts (*p* = 0.0004; Table 5). In addition, for the heterosexual group, multiple logistic regression showed that the presence of perceived social ageism was significantly associated with the risk to perceive ageism toward the sexuality of elderly people (OR = 3; 95% CI 1.13 to 7.93; *p* = 0.02). For the LGB group, multiple logistic regression showed that the presence of occasions of sociality protected from the risk of having perceived ageism toward sexuality in the elderly (OR = 0.10; 95% CI 0.02 to 0.54; *p* = 0.007). Thus, LGB people who have occasions of sociality have a reduced probability of experiencing perceived ageism toward sexuality (Supplementary Tables S7 and S8).

### 3.4. Dysfunctional Beliefs toward Ageing and Sexuality

With regard to dysfunctional beliefs about ageing, univariate logistic regression analysis showed a statistically significant difference between the two study groups (*p* = 0.004) (Table 6). In addition, for heterosexuals, multiple logistic regression showed that the perception of the reduction in the quality of sexual intercourse was significantly associated with the risk of having dysfunctional beliefs toward ageing (OR = 15.93; 95% CI 5.60 to 45.34; *p* < 0.0001). For the LGB group, multiple logistic regression showed that the perception of the reduction in the quality of sexual intercourse (OR = 60,84; 95% CI 6.34 to 583.88; *p* = 0.0004) and the experience of loneliness (OR = 9.87; 95% CI 1.01 to 96.22; *p* = 0.04) were significantly associated with dysfunctional beliefs toward ageing (Supplementary Tables S9 and S10).

Table 7 presents data about dysfunctional beliefs toward sexuality. Interestingly, dysfunctional beliefs about the sexuality of the elderly were significantly more present among heterosexuals (*p* = 0.0001). In addition, for both heterosexuals and LGB people multiple logistic regression showed that none of the selected variables significantly impacted on this difference.

## 4. Discussion

To the best of our knowledge, this is the first study evaluating the role of ageism and dysfunctional beliefs in the sexual life of elderly people, comparing these aspects among heterosexuals and LGB people. We found some interesting differences between the two study groups in terms of sexual activity, ageism related to sexuality, and dysfunctional beliefs related to sexual behavior.

With regard to demographic distributions, the two groups significantly differed in terms of gender distribution, relational status, and co-habitation status; indeed, within the LGB group, the respondents were more likely to be male, to be unmarried, and to live alone. This result is consistent with the sociocultural conditions of the LGB community: female homosexuals and bisexuals are more likely to be invisible and not declared. As regards relational and co-habitation status, it is of note that in Italy, civil unions among non-heterosexual people have only been legally recognized since 2016.

With regard to sexual activity, we found that LGB people show a higher frequency of sexual activity (masturbation and sexual intercourse) than heterosexual individuals and an increase in the quality of sexual activity. The presence of sexual dysfunctions, on the contrary, was not statistically significantly different between the two study groups: only 5.5% and 8% of LGB and heterosexual individuals, respectively, declared to suffer from sexual dysfunctions. Our results are partially in contrast with those from Brennan-Ing et al.; the authors found that older LGB adults were as likely to remain sexually active as older heterosexuals; moreover, they found that gay and bisexual men were more likely than heterosexual men to report some sexual problems [43]. Our study findings suggest that heterosexuals’ sexual activity is negatively influenced by perceived social ageism and by dysfunctional beliefs toward ageing. With regard to LGBs’ sexual activity, on the contrary, concrete aspects of daily life, such as co-habitation status and occasions of sociality, play a more significant role. This is an interesting result, suggesting a bigger influence of ageism and internalized aspects of ageism on sexual activity for heterosexual people than for LGB people. Future studies are needed to confirm these data.

It is well known that sexual activity positively correlates with general and psychological well-being [44,45,46]. This becomes yet more significant in the elderly period when persons are physiologically exposed to a higher risk of developing several diseases. The literature evidence shows that in later years, heterosexual people remain sexually active [47,48,49]. Unfortunately, data about the sexual life of elderly LGBTQ+ individuals are sparse or lacking. This domain of sexual medicine and psycho-sexology is still understudied, and several methodological problems tend to persist [50]. Some studies have shown no significant differences in the frequency of sexual intercourse among elderly heterosexuals and homosexuals [27,51], with significantly lower levels of sexual satisfaction reported by homosexual people [27]. Our data, on the contrary, suggested that the frequency of sexual intercourse is higher in the LGB group, as compared with the heterosexual one. A possible explanation for these data could be related to the necessity to manage double minority stress, consisting of the co-existence of stress due to sexual orientation, and to interiorized stress in relation to ageism and the sexuality of the elderly, also with occasions of sociality. Thus, through sexual activity and with occasions of sociality, the elderly could reduce their distress and, as for the generalized interiorized stigma, could manage the interiorized stigma about the sexual life of elderly LGB individuals [52]. The literature reports that sex can be used as a coping strategy to ward off stress, especially among vulnerable populations and sex offender populations [53,54].

As a second result, we found that social ageism toward ageing is perceived in an equal manner by heterosexual and LGB elderly individuals. This is not surprising given that society tends to associate ageing with negative beliefs, and that they tend to be perpetuated from the beginning of the social perception process [3,19]. On the contrary, social ageism associated with sexuality was more prevalent among LGB people than among heterosexual people; specifically, LGB individuals significantly perceived more absence of representation of sexuality in the elderly than heterosexual individuals. These results may mirror the double invisibility LGB older adults face in actual society, as being both older adults and belonging to a sexual minority. Specifically, we can hypothesize that social ageism associated with sexuality captures a core identity aspect of LGB people, as well as a stigmatized one; therefore, LGB older adults may perceive this aspect as a more salient and relevant aspect than heterosexual older adults and try to manage it with the higher prevalence of occasions of sociality. LGB people are likely to have suffered recurrent experiences of discrimination and prejudice during their lifespan, which may contribute to a higher sensitivity in regard to such stereotypical representations [55]. Of note, the literature reports that many LGB older adults report that their sexual orientation makes ageing more difficult and that they tend to conceal this identity aspect, contributing to the risk of a vicious circle between the perception of invisibility and social invisibility [56]. Future studies should evaluate sexuality in elderly people considering this social invisibility.

Of note, the results from the multiple logistic regression analysis confirmed the results regarding sexual activity; indeed, for the heterosexual group, the presence of perceived social ageism significantly increased the risk of having perceived ageism toward the sexuality of elderly people. For the LGB group, the presence of occasions of sociality significantly protected them from the risk of having perceived ageism toward sexuality in the elderly. These results provide interesting information that can be used to implement specific interventions to promote healthy and successful ageing, targeted for groups, also according to sexual orientation.

On the other hand, with regard to dysfunctional beliefs about sexuality [17], we found that heterosexual people endorse significantly more dysfunctional beliefs than LGB individuals. LGB older adults, because of their previous experiences as a stigmatized sexual minority, could better manage certain aspects related to sexual stereotypes, having developed personal skills to maintain personal beliefs and being less prone to interiorize dysfunctional ones. Consistent with the crisis competence theory, individuals who have one stigmatized identity develop skills to cope with stigmatization, that can be used to cope with other experiences of stigmatization [57]. Consistent with this, some previous studies have highlighted that a certain proportion LGBT adults reported that their sexual orientation, as a sexual minority, helped them prepare for ageing, endowing them with greater resilience [58,59]. This is an interesting result, that suggests the need to focus not only on risk factors but also on protective factors for LGB older adults’ sexual health [60]. Moreover, as suggested by Slevin and Mowery, understanding LGB sexuality requires a life course lens [61]. Specific to the actual community of LGB older adults, it is of note that they represent a specific age cohort; indeed, during their life course, actual LGB elders have experienced significant societal changes due to the civil rights movements that have taken place in the recent decade; all of these changes may have positively impacted on LGB older adults’ quality of life and sexual life as well.

This study shows some limitations, which make our findings not completely representative of the general population of heterosexual and LGB people. First, the participants of the LGB sample were enrolled with a specific association dedicated to LGB people. Thus, it is reasonable to suppose the presence of selection bias. However, given the significant invisibility that still characterizes LGB older adults, this recruitment method allowed us to reach this target population. Second, the online fulfillment of the survey has limited access to those elderly who are able to connect online; this aspect could influence the sample’s representativeness. Third, the adoption of non-standardized measures for the evaluation of study purposes may represent another limit. On the other hand, as a pilot study, this research was useful to better understand the role of ageism toward the sexuality of elderly heterosexuals and LGB individuals, giving an address to future studies that will develop specific tools to evaluate these aspects. Finally, the present study used exclusively quantitative data; although this methodology offers a general picture, it does not allow us to consider the personal meanings associated with ageism and sexuality in ageing. Future studies could incorporate both quantitative and qualitative methodology, to provide a deeper understanding of these topics.

Based on all these considerations, we cannot make firm conclusions with respect to the negative consequences of sexual ageism and sexual dysfunctional beliefs on LGB and heterosexual people, respectively. However, overall, the study findings highlight the significance of examining sexual orientation to understand experiences of sexuality in the ageing of the growing older population. This study is the first attempt in evaluating in which manner the co-existence of two forms of hypothetical and subjective stressors (elderly age and non-normative sexual orientation) may bear on the sexual life of individuals. Future studies with a stronger methodology will further suggest the need for social education on sexual ageism.

## 5. Conclusions

The findings of this pilot study suggest that ageism and dysfunctional beliefs toward ageing and sexuality impact differently on older adults’ sexual life and with different patterns of associations, according to sexual orientation. Specifically, heterosexual older adults seem to be more vulnerable to the influence of ageism and of internalized aspects of ageism on sexual activity; in our samples, heterosexuals were more prone to endorse dysfunctional beliefs about sexuality; moreover, heterosexuals’ sexual activity was more negatively affected by social ageism toward ageing and more intensely associated with ageism toward the sexuality of elderly people. LGB older adults, on the contrary, seem to be more resilient toward these aspects; LGB are more likely to experience an improvement in the quality of their sexual life; concrete aspects, such as social isolation and occasions of sociality, are suggested to play a role in LGB older adults’ sexual life. To conclude, the study findings highlight the significance of examining sexual orientation to understand experiences of sexuality in the ageing of the growing older population. Renewed socio-educational efforts based on these data are clearly needed.

## Figures and Tables

**Table 1:** Demographics.

Variables	Heterosexual Group (*n* = 104)	Control Group (*n* = 111)	*p*
Age (mean; 95% CI for the mean)	69; 67.8–70.6	64; 63.2–66.6	0.0001
Sex assigned at birth (*n*; %)			
Male	49 (47.1%)	90 (81%)	<0.0001
Female	55 (52.8%)	21 (18.9%)	<0.0001
Gender Identity (*n*; %)			
Cisgender	104 (100%)	103 (92.8%)	0.0001
Non binary	0 (0%)	2 (1.8%)	<0.0001
Transgender MtF	0 (0%)	6 (5.4%)	<0.0001
Transgender FtM	0 (0%)	0 (0%)	/
Variables	Final group	Final group	*p*
	Heterosexual group	LGB group	
	(*n* = 104)	(*n* = 103)	
Relational status (*n*; %)			
Celibate/nubilate	9 (8.6%)	61 (59.2%)	<0.0001
Civil marriage	3 (2.8%)	27 (26.2%)	<0.0001
Marriage/cohabiting	64 (61.5%)	5 (4.8%)	<0.0001
Separated	6 (5.7%)	4 (3.8%)	0.72
Divorced	14 (13.5%)	4 (3.8%)	0.02
Widowed	8 (7.7%)	2 (1.8%)	0.09
Whit whom do you live? (*n*; %)			
Alone	30 (28.8%)	53 (51.4%)	0.001
With the partner	49 (47.1%)	36 (34.9%)	0.1
With the partner and children	20 (19.2%)	3 (2.9%)	0.0004
Alone with children	3 (2.8%)	2 (1.9%)	0.9
With other persons	2 (1.9%)	9 (8.7%)	0.9
Religion (*n*; %)			
Believer and practicing	17 (16.3%)	6 (5.8%)	0.02
Believer and not practicing	39 (37.5%)	25 (24.2%)	0.05
Not believer	48 (46.1%)	72 (69%)	0.001

**Table 2:** Physical and psycho-social well-being.

Variables	Heterosexual Group (*n* = 104)	LGB Group (*n* = 103)	*p*
Subjective perception of health (*n*; %)			
Bad (1)	6 (5.8)	12 (11.7)	0.2
Good (2)	84 (80.8)	83 (80.6)	0.88
Optimal (3)	14 (13.5)	8 (7.8)	
Participation to social activities (*n*; %)			
Never (0)	2 (1.9)	10 (9.7)	
Two-three times at month (1)	20 (19.2)	36 (35)	
One time at week (2)	12 (11.5)	15 (14.6)	
Two times at week (3)	26 (25)	20 (19.4)	
Three-six times at week (4)	35 (33.7)	20 (19.4)	0.02
Each day (5)	9 (8.7)	2 (1.9)	
To experience depression in the last 12 months (*n*; %)			
Always (4)	1 (22.1)	1 (1)	
Often (3)	15 (33.7)	16 (15.5)	
Sometimes (2)	30 (28.8)	42 (40.8)	
Rarely (1)	35 (14.4)	33 (32)	0.09
Never (0)	23 (1)	11 (10.7)	
To experience loneliness in the last 12 months (*n*; %)			
Always (4)	1 (1)	3 (2.9)	
Often (3)	9 (8.7)	18 (17.5)	
Sometimes (2)	22 (21.2)	32 (31.1)	0.09
Rarely (1)	27 (26)	25 (24.3)	0.14
Never (0)	45 (43.3)	25 (24.3)	

**Table 3:** Univariate logistic regression with the variable sexual activity in heterosexual and LGB older adults.

Variables	Heterosexual Group (*n* = 104)	LGB Group (*n* = 103)	Odds Ratio	95% CI	*p*
Frequency of sexual intercourse is					
increased (*n*; %)					
Completely agree (0)	2 (2.1)	88 (85.4)	0.126	0.028–0.567	0.0009
Disagree at all (1)	93 (97.9)	15 (14.6)			
Frequency of masturbation is increased (*n*; %)					
Completely agree (1)	11 (10.6)	29 (28.2)	3.313	1.552–7.071	0.001
Disagree at all (0)	93 (89.4)	74 (71.8)			
Quality of sexual intrecourse is increased (*n*; %)					
Completely agree (1)	18 (19.1)	36 (35)	3.116	1.567–6.198	0.001
Disagree at all (0)	76 (80.9)	67 (65)			
Presence of sexual dysfunctions (*n*; %)					
No	95 (91.8)	97 (94.3)	0.722	0.197–2.640	0.77
Yes	4 (8.2)	6 (5.8)			

**Table 4:** Univariate logistic regression of the variable perceived social ageism toward ageing between the two study groups.

Variables	Heterosexual Group (*n* = 104)	LGB Group (*n* = 103)	Odds Ratio	95% CI	*p*
Image society gives to aging people (*n*; %)					
Positive/ Neither positive nor negative (0)	59 (56.7)	49 (47.6)	0.692	0.40–1.196	0.18
Negative (1)	45 (43.3)	54 (52.4)			
Do you perceive yourself represented by the image society has of the elederly people? (*n*; %)					
Enough/very (0)	14 (13.5)	12 (11.7)	0.847	0.371–1.933	0.69
For nothing/little (1)	90 (86.5)	91 (88.3)			
Perceived ageism toward aging (*n*; %)					
Yes (0)	60 (57.7)	56 (54.4)	1.144	0.66–1.982	0.63
No (1)	44 (42.3)	47 (45.6)			

**Table 5:** Univariate logistic regression of the variable perceived social ageism toward sexuality of elderly people between the two study groups.

Variables	Heterosexual Group (*n* = 104)	LGB Group (*n* = 103)	Odds Ratio	95% CI	*p*
Society represents the sexuality of the elderly as non-existing (*n*; %)					
Disagree at all (0)	62 (59.6)	36 (35)	2.74	1.56–4.83	0.0004
Agree at all (1)	42 (40.4)	67 (65)			

**Table 6:** Univariate logistic regression of the variable dysfunctional beliefs about the elderly between the two study groups.

Variables	Heterosexual Group (*n* = 104)	LGB Group (*n* = 103)	Odds Ratio	95% CI	*p*
You worry about the idea of getting old (*n*; %)					
For nothing/little (0)	61 (58.7)	40 (38.8)	0.447	0.256–0.78	0.004
Enough/very (1)	43 (41.3)	63 (61.2)			

**Table 7:** Univariate logistic regression of the variable dysfunctional beliefs about the sexuality of the elderly between the two study groups.

Variables	Heterosexual Group (*n* = 104)	LGB Group (*n* = 103)	Odds Ratio	95% CI	*p*
Sexuality is important at all life stages (*n*; %)					
Completely agree (0)	102 (98.1)	102 (99)	1.98	0.176–22.18	0.57
Disagree at all (1)	2 (1.9)	1 (1)			
Sexuality is not about older people (*n*; %)					
Completely agree (1)	9 (8.7)	4 (3.9)	2.344	0.698–7.87	0.167
Disagree at all (0)	95 (91.3)	99 (96.1)			
Dysfunctional beliefs about sexuality of elderly (*n*; %)					
Absent (0)	70 (67.3)	93 (90.4)	4.565	2.114–9.86	0.0001
Present (1)	34 (32.7)	10 (9.6)			

## Data Availability

Data will be available upon reasonable request to the authors.

## Supplementary Materials

The following supporting information can be downloaded at: https://www.mdpi.com/article/10.3390/healthcare11040459/s1. Table S1: Multiple stepwise logistic regression analysis with “Frequency of sexual intercourse in the last 12 months in heterosexual older adults” as dependent variable; Table S2: Multiple stepwise logistic regression analysis with “Frequency of sexual intercourse in the last 12 months in LGB older adults” as dependent variable; Table S3: Multiple stepwise logistic regression analysis with “Frequency of masturbation in heterosexual older adults” as dependent variable; Table S4: Multiple stepwise logistic regression analysis with “Frequency of masturbation in LGB older adults” as dependent variable; Table S5: Multiple stepwise logistic regression analysis with “Quality of sexual activity improved in heterosexual older adults” as dependent variable; Table S6: Multiple stepwise logistic regression analysis with “Quality of sexual activity improved in LGB older adults” as dependent variable; Table S7: Multiple stepwise logistic regression analysis with “Society represents sexuality of elderly as non-existing in heterosexual older adults” as dependent variable; Table S8: Multiple stepwise logistic regression analysis with “Society represents sexuality of elderly as non-existing in LGB older adults” as dependent variable; Table S9: Multiple stepwise logistic regression analysis with “Dysfunctional beliefs in heterosexual older adults” as dependent variable; Table S10: Multiple stepwise logistic regression analysis with “Dysfunctional beliefs in LGB older adults” as dependent variable.
