# Supplementary material for: “I’m Too Old for That”: The Role of Ageism and Sexual Dysfunctional Beliefs in Sexual Health in a Sample of Heterosexual and LGB Older Adults: A Pilot Study
Source: Healthcare (Basel). 2023 Feb 5;11(4):459. doi: 10.3390/healthcare11040459 (PMC9957165; doi:10.3390/healthcare11040459)
Supplement: Supplementary file 1 [file healthcare-11-00459-s001.zip › healthcare-2167580-supplementary.pdf]

Supplementary materials

Table S1: Multiple stepwise logistic regression analysis with "Frequency of sexual intercourse in the last 12 months in heterosexual older adults" as dependent variable

Independent variables	Regression coefficient	P-value	SE	OR	95% CI
Depressive mood	-1.082	0.04	0.549	0.338	0.115 to 0.993
Quality of sexual intercourse improved	1.257	0.02	0.576	3.517	1.136 to 10.891

Table S2: Multiple stepwise logistic regression analysis with "Frequency of sexual intercourse in the last 12 months in LGB older adults" as dependent variable

Independent variables	Regression coefficient	P-value	SE	OR	95% CI
Cohabitation	2.173	0.001	0.658	8.79	2.416 to 31.979
Participation to social activities	2.872	0.0004	0.81	17.677	3.613 to 86.485

Table S3: Multiple stepwise logistic regression analysis with "Frequency of masturbation in heterosexual older adults" as dependent variable

Independent variables	Regression coefficient	P-value	SE	OR	95% CI
Ageism toward elderly	1.922	0.01	0.822	6.838	1.364 to 34.269

Table S4: Multiple stepwise logistic regression analysis with "Frequency of masturbation in LGB older adults" as dependent variable

Independent variables	Regression coefficient	P-value	SE	OR	95% CI
Ageism toward elderly	1.516	0.001	0.488	4.554	1.75 to 11.85
Cohabitation	-1.08	0.03	0.524	0.336	0.12 to 0.94

Table S5: Multiple stepwise logistic regression analysis with "Quality of sexual activity improved in heterosexual older adults" as dependent variable

Independent variables	Regression coefficient	P-value	SE	OR	95% CI
Dysfunctional beliefs	-2.514	0.01	1.054	0.08	0.01 to 0.63

Table S6: Multiple stepwise logistic regression analysis with "Quality of sexual activity improved in LGB older adults" as dependent variable

Independent variables	Regression coefficient	P-value	SE	OR	95% CI
Participation to social activities	2.294	0.0001	0.583	9.916	3.157 to 31.15

Table S7: Multiple stepwise logistic regression analysis with "Society represents sexuality of elderly as non-existing in heterosexual older adults" as dependent variable

Independent variables	Regression coefficient	P-value	SE	OR	95% CI
Ageism toward elderly	1.09	0.02	0.496	3	1.134 to 7.931

Table S8: Multiple stepwise logistic regression analysis with "Society represents sexuality of elderly as non-existing in LGB older adults" as dependent variable

Independent variables	Regression coefficient	P-value	SE	OR	95% CI
Participation to social activities	-2.21	0.007	0.822	0.10	0.021 to 0.548

Table S9: Multiple stepwise logistic regression analysis with "Dysfunctional beliefs in heterosexual older adults" as dependent variable

Independent variables	Regression coefficient	P-value	SE	OR	95% CI
Decreased quality of sexual activity	2.768	<0.0001	0.533	15.937	5.6 to 45.34

Table S10: Multiple stepwise logistic regression analysis with “Dysfunctional beliefs in LGB older adults” as dependent variable

Independent variables	Regression coefficient	P-value	SE	OR	95% CI
Decreased quality of sexual activity	4.108	0.0004	1.153	60.842	6.34 to 583.88
To perceive loneliness	2.29	0.048	1.161	9.879	1.014 to 96.228
